# Directed Differentiation of Embryonic Stem Cells Into Cardiomyocytes by Bacterial Injection of Defined Transcription Factors

**DOI:** 10.1038/srep15014

**Published:** 2015-10-09

**Authors:** Fang Bai, Chae Ho Lim, Jingyue Jia, Katherine Santostefano, Chelsey Simmons, Hideko Kasahara, Weihui Wu, Naohiro Terada, Shouguang Jin

**Affiliations:** 1 State Key Laboratory of Medicinal Chemical Biology and Colleges of Pharmacy and Life Sciences, Nankai University, Tianjin, China; 2 Department of Molecular Genetics and Microbiology College of Medicine, University of Florida, Gainesville, FL 32610; 3 Department of Pathology College of Medicine, University of Florida, Gainesville, FL 32610; 4 Department of Mechanical & Aerospace Engineering College of Engineering, University of Florida, Gainesville, FL 32611; 5 Department of Physiology and Functional Genomics, University of Florida, College of Medicine, Gainesville, Florida, United States of America

## Abstract

Forced expression of defined transcriptional factors has been well documented as an effective method for cellular reprogramming or directed differentiation. However, transgene expression is not amenable for therapeutic application due to potential insertional mutagenesis. Here, we have developed a bacterial type III secretion system (T3SS)-based protein delivery tool and shown its application in directing pluripotent stem cell differentiation by a controlled delivery of transcription factors relevant to early heart development. By fusing to an N-terminal secretion sequence for T3SS-dependent injection, three transcriptional factors, namely Gata4, Mef2c, and Tbx5 (abbreviated as GMT), were translocated into murine embryonic stem cells (ESCs), where the proteins are effectively targeted to the nucleus with an average intracellular half-life of 5.5 hours. Exogenous GMT protein injection activated the cardiac program, and multiple rounds of GMT protein delivery significantly improved the efficiency of ESC differentiation into cardiomyocytes. Combination of T3SS-mediated GMT delivery and Activin A treatment showed an additive effect, resulting in on average 60% of the ESCs differentiated into cardiomyocytes. ESC derived cardiomyocytes displayed spontaneous rhythmic contractile movement as well as normal hormonal responses. This work serves as a foundation for the bacterial delivery of multiple transcription factors to direct cell fate without jeopardizing genomic integrity.

Forced expression of transcription factors (TFs) has been well documented as an effective method for directing both cellular differentiation and reprogramming^1^,^2^,^3^, and this approach has relied heavily on the use of transgene expression to alter endogenous lineage-specific gene expression patterns. Given the potential for insertional and recombination-mediated mutagenesis associated with such DNA-based techniques, cells derived from the use of such methods have limited clinical applicability. To overcome these shortcomings, a transient non-DNA or non-viral approach is highly desirable. Protein delivery serves is a safe alternative and there are a number of well-known protein delivery technologies, the best one being fusion to cell penetrating peptide derived from Tat protein of retrovirus^4^,^5^, however, they are limited by the need for protein purification and low targeting efficiency. Development of a simple and efficient system for the introduction of TFs is required to meet an emerging need which is quite apparent from recent studies.

*Pseudomonas aeruginosa* is a common gram-negative opportunistic human pathogen which injects proteineous exotoxins directly into host cells via a type III secretion system (T3SS)^6^. The T3SS is a complex, needle-like structure on bacterial surface, responsible for the secretion of four known exotoxins: ExoS, ExoT, ExoY or ExoU^7^. Of these exotoxins, ExoS is the best characterized for its functional domains, with its N-terminal sequence serving as a signal for injection through the type III needle apparatus^8^. Since this naturally occurring protein injection machinery does not involve bacteria entering the host cells or DNA integration, *P. aeruginosa* is ideal for the delivery of exogenous proteins into mammalian cells. Previously, we have fused various lengths of the ExoS N-termini with Cre recombinase for injection into mammalian cells and found that N-terminal 54 amino acids (ExoS_54_) were optimal for the delivery of the exogenous Cre protein. The injected Cre protein was efficiently targeted to nucleus and mediated LoxP site-dependent DNA recombination^9^. Similarly, we have effectively delivered a pair of transcription activator-like effector nuclease (TALEN) proteins fused with the ExoS_54_ into HeLa cells, achieving site specific DNA cleavage on intended locus^10^. Additionally, a muscle specific master transcription factor MyoD was successfully injected into mouse embryonic fibroblasts, converting them into muscle cells^11^.

Cardiovascular disease is a leading cause of death worldwide^12^,^13^,^14^. The limited capability of heart tissue to regenerate has prompted methodological developments for creating *de novo* cardiomyocytes, both *in vitro* and *in vivo*. Beyond uses in cell replacement therapy, patient-specific cardiomyocytes may find applications in drug testing, drug discovery, and disease modeling. Mammalian cardiomyocytes can be generated from embryonic stem cells (ESCs) *in vitro* by a variety of methods^15^. Most available protocols involve growth factor-directed differentiation of monolayers or embryoid bodies in a variety of serum-free defined media^16^. Recently, an alternative source of *de novo* cardiomyocytes was demonstrated, deriving from fibroblasts via “direct reprogramming” or also known as transdifferentiation^17^,^18^. Several groups have reported the *in vitro* or *in vivo* reprogramming of mouse fibroblasts to cardiomyocyte-like cells by various combinations of core cardiac developmental transcription factors^19^,^20^,^21^,^22^,^23^,^24^.

In this study, we further optimized the T3SS-based protein delivery system for its application in pluripotent stem cells, testing out on directed embryonic stem cell (ESC) differentiation into cardiomyocytes (CMs) by simultaneous injection of multiple transcriptional factors that are relevant to cardiomyocyte development. During early heart development, the GMT transcription factors Gata4, Mef2c, and Tbx5 (short as GMT) interact with one another to co-activate cardiac gene expression, such as *Actc1* (alpha cardiac actin), *cTnT*, (cardiac troponin T), and *MYH6* (α-myosin heavy chain, also called αMHC), and promote cardiomyocyte differentiation^25^,^26^,^27^,^28^,^29^. It has previously been reported that a combined overexpression of the GMT *via* viral vector can successfully reprogram mouse cardiac and dermal fibroblasts into cardiomyocyte-like cells^19^,^20^. Accordingly, we have developed a bacterial T3SS-based TFs delivery tool and shown its capability to efficiently tanslocate GMT into mouse ESCs. Using this method, we demonstrate that GMT proteins are sufficient to activate the expression of cardiac specific genes and promote ESC-derived cardiomyocytes (ESC-CMs) differentiation. Interestingly, mesodermal inducer Activin A showed an additive effect on the GMT injection mediated promotion of ESC-CMs differentiation, enabling us to achieve 10 folds higher efficiency of ESC-CMs differentiation than that of spontaneous differentiation. We also demonstrate that T3SS-based protein delivery system is highly controllable, in terms of injection dose, order and duration, opening up the possibility of using this novel technology to direct induced pluripotent stem cell differentiation to desired cell types for various biomedical applications.

## Results

### Development of a novel protein delivery tool based on T3SS of P. aeruginosa suitable for ES cells

**Figure 1:**
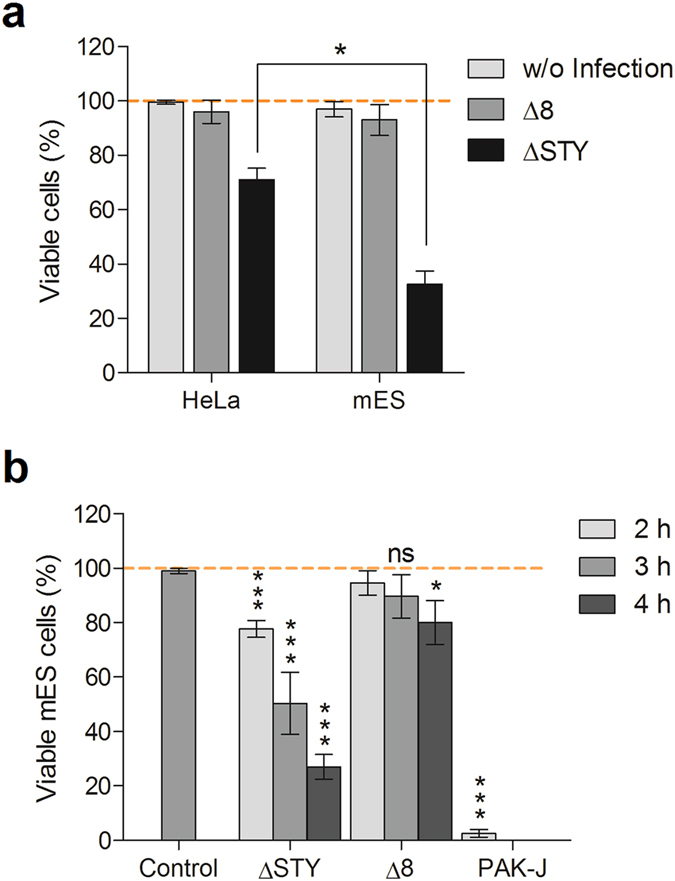
Cytotoxicity assay of various *P. aeruginosa* strains. (**a**) HeLa cells and mES cell line R1 were infected with indicated strains for 3 h at MOI of 100, and cells that remaine adhered were counted. ΔSTY, deleted of all three type III secreted toxins; Δ8, deleted of 8 virulence genes. (**b**) mES cells were infected with the indicated strains for 2, 3, 4 h at an MOI of 100, and cells that remaine adhered were counted. PAK-J, wild-type; Control, without bacterial infection. Data represent means of three replicate experiments. Error bars represent SD. *P < 0.05, *** *P* < 0.001.

Previously, by fusing a short N-terminal secretion sequence (54aa peptide) for T3SS-dependent injection, we have successfully delivered various functional proteins into mammalian cells using *P. aeruginosa* strain ΔSTY, which is an engineered low cytotoxicity strain, deleted of three well-known endogenous toxin genes (*exoS*, *exoT* and *exoY*) but maintains a high type III secretion capacity (Table 1)^9^,^10^,^11^. However, it shows an obvious cytotoxicity on HeLa cells after co-incubation for 3 h at MOI of 100, with approximately 30% of the HeLa cells become rounded and lifted^9^. Pluripotent stem cells (like embryonic stem cells) are much more sensitive to the bacterial cytotoxicity than somatic cells (Fig. 1a). To use *P. aeruginosa* as a protein delivery vehicle for pluripotent stem cells, the bacterial cytotoxicity needs to be decreased further. To this end, strain ΔSTY was further deleted of genes implicated in the bacterial virulence, including a nucleoside diphosphate kinase gene (*ndk*)^30^, a structural gene for the type II secretion system (*xcpQ*)^31^, genes for quorum sensing signal synthesis (*lasI* and *rhlI*)^32^,^33^ and an inhibitor gene for the type III secretion (*popN*)^34^,^35^, resulting in a strain deleted of 8 genes in total, thus designated the resulting strain as Δ8 (Table 1).

**Figure 2:**
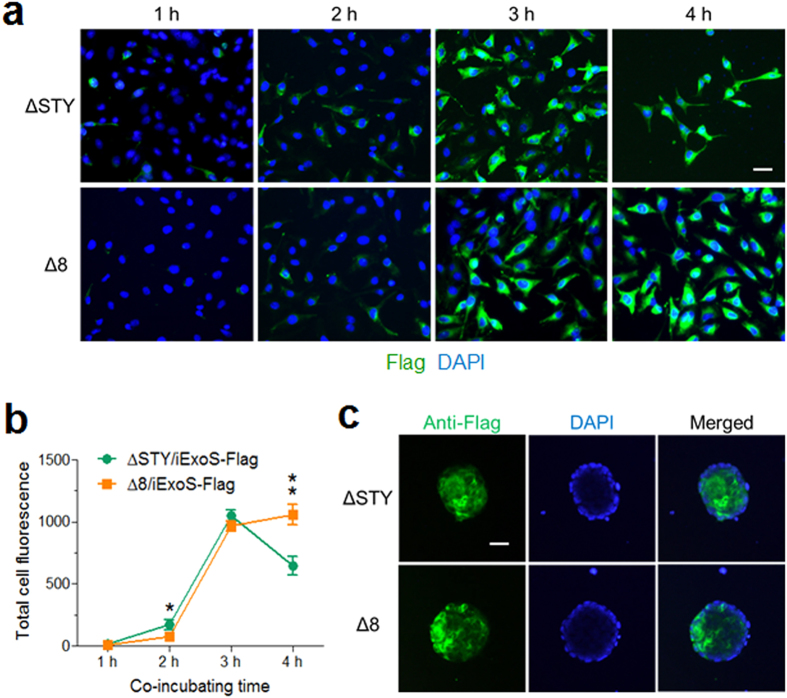
T3SS-dependent protein injection capability of various P. aeruginosa strains. (**a**) Immunohistochemistry of HeLa cells following infection by ΔSTY/piExoS-Flag or Δ8/piExoS-Flag for 1, 2, 3, 4 h at MOI of 50. Cells were stained with anti-Flag antibody and nuclei with DAPI stain. (**b**) Quantification of anti-Flag immunofluorescence staining intensity within HeLa cells as shown in (**a**). Data represent means of three replicative experiments. Error bars represent SD. *P < 0.05, ** *P* < 0.01. (**c**) Immunohistochemistry of mES cells following infection by ΔSTY/piExoS-Flag or Δ8/piExoS-Flag and for 3 h at MOI of 50. Cells were stained with anti-Flag antibody; nuclei with DAPI stain. Bar = 50 μm.

The cytotoxicity of strain Δ8 was compared to that of the wild-type strain PAK-J and ΔSTY. Mouse ES cells were infected by these three strains at MOI 100 for various time and the number of cells that remain adhered to tissue culture plates were counted. As the results shown in Fig. 1b, there was no significant cytotoxicity 3 h post-infection with the Δ8, although by 4 h post-infection, 80% of the cells remain adhered to the plate, whereas incubation with the ΔSTY resulted in only 30% of cells still adhering and none with that of wild type PAK-J. Since maximum protein injection is normally achieved by 3 hours of infection, the Δ8 seems an appropriate strain for protein delivery. To evaluate the protein injection capability of the Δ8, a fusion of catalytically inactive ExoS with Flag-tag (iExoS-Flag) was injected into HeLa and mES cells by either ΔSTY or Δ8. As shown in Fig. 2a,b, the levels of injected iExoS-Flag fusion in HeLa cells by both strains were comparable at MOI = 50 for 3 hours. However, about half of the cells were lifted and lysed after infection with ΔSTY for 4 h, while no obvious cell lifting was observed following infection by Δ8. For mouse ES cells, iExoS-Flag fusion was efficiently injected into the mESCs by Δ8 at MOI of 50 within 3 hours of infection time (Fig. 2c). These results demonstrate that the new ∆8 strain indeed has a much lower cytotoxicity than ∆STY, yet maintains a high type III secretion capacity.

**Figure 3:**
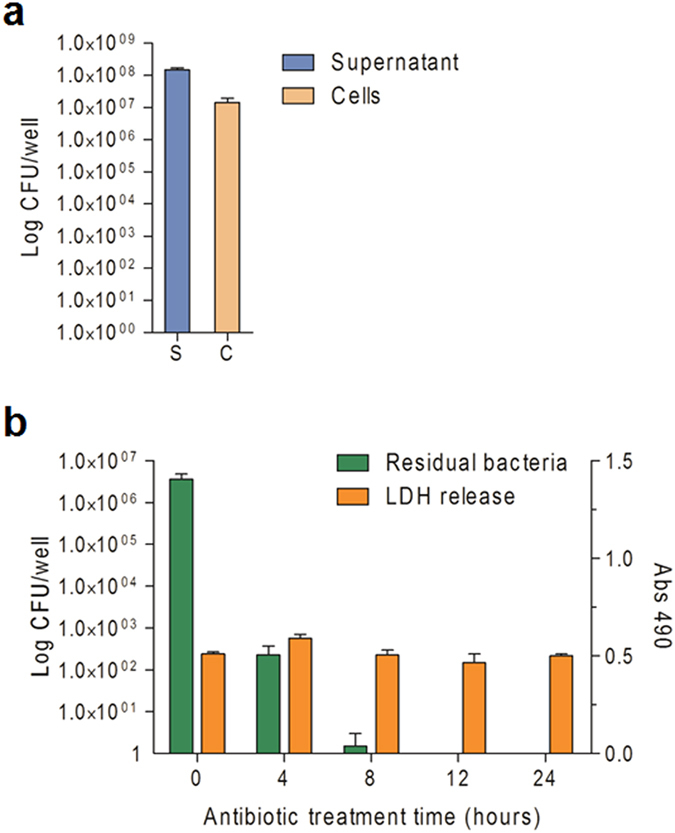
Elimination of residual bacteria by antibiotic treatment. (**a**) mES cell line R1 was infected with ∆8 at MOI of 100 for 3 hours. Supernatants and adherent ES cells of each well were collected and serially diluted, then plated on LB-agar plates to enumerate the bacterial cell number (cfu/well) of planktonic bacteria (S) and bacteria attached to the mES cells (C), respectively. (**b**) Infection was terminated by washing cells with PBS and continuous growth of the mES cells on culture medium containing 20 μg/mL ciprofloxacin. After antibiotic treatment (time 0 h), 50-μL cell culture supernatant per well was used for LDH release assay. At the same time, mES cell colonies were scraped and lysed by 0.2% Triton-X100, the lysates were serially diluted and plated on LB-agar plates to calculate the residual bacterial numbers (cfu/well).

Elimination of the bacterial cells after the completion of protein delivery is another major concern. Following 3 h of infection by Δ8 at MOI 100, majority of the bacterial cells (90%) remain floating and can easily be removed by a washing step, but about 10% of input bacterial cells become attached to the ES cells (Fig. 3a). To eliminate the residual adhering bacterial cells, the ES cells were sub-cultured in medium containing 20 μg/mL ciprofloxacin which is an effective antibiotic for *P. aeruginosa*. ES cells were scraped off from the plate at various time points and viable bacterial cells were enumerated by plating on L-agar medium. As the results shown in Fig. 3b and Supplementary Fig. 1, the number of viable bacterial cells gradually deceased, with no detectable bacterial cells or bacterial housekeeping gene by 12 hours. Within the same time frame, treatment of ES cells with the 20 μg/mL ciprofloxacin alone showed no cytotoxic effect demonstrated by the lactate dehydrogenase (LDH) release analysis.

### Bacterial production and injection of transcription factors into ES cells

**Figure 4:**
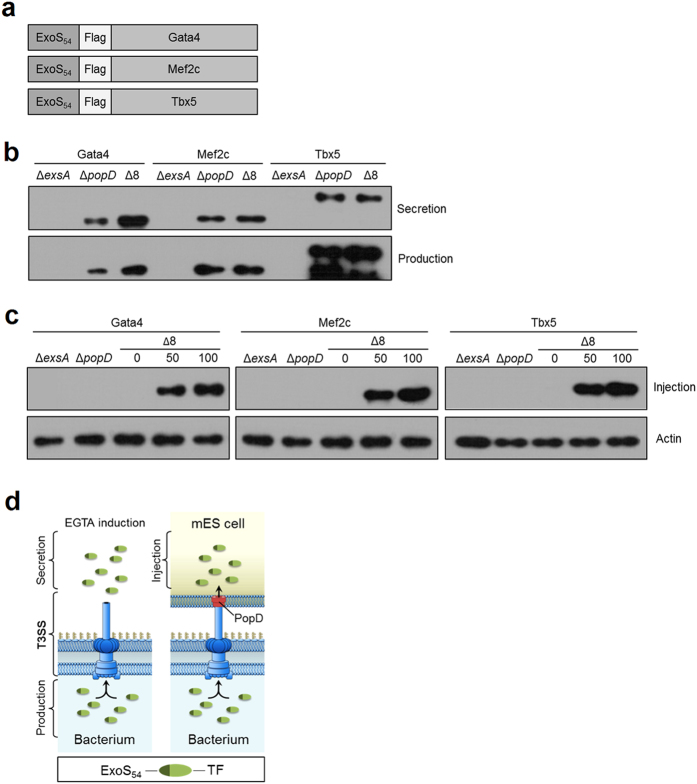
Bacterial T3SS mediated production and injection of TF proteins into mES cells. (**a**) Schematic representation of plasmids encoding the ExoS_54F_-Gata4, ExoS_54F_-Mef2c and ExoS_54F_-Tbx5 fusions with a Flag-tag fused in the middle. (**b**) Δ*exsA*, Δ*popD* and Δ8 strains with plasmids expressing ExoS_54F_-Gata4, ExoS_54F_-Mef2c or ExoS_54F_-Tbx5 fusion. Each strain was examined for the ability to produce and secrete the fusion protein by anti-Flag immunoblot of the bacterial pellets and culture supernatant. (**c**) mESCs were infected with each strain at indicated MOI for 3 hours, lysed and examined for protein injection by anti-Flag immunoblot. (**d**) Schematic representation of T3SS-dependent protein production and secretion or injection (protein translocation).

An expression vector pExoS_54_F was constructed by cloning a DNA fragment containing the *P. aeruginosa* exotoxin ExoS promoter and N-terminal T3SS secretion signal (ExoS_54_), followed by a Flag-tag, into the multiple cloning site (MCS) of *E. coli*-*Pseudomonas* shuttle vector pUCP19 (Table 1 and Supplementary Fig. 2). Three transcriptional factors (TFs), Gata4, Mef2C and Tbx5 were cloned into the pExoS_54_F, generating in-frame fusions behind the ExoS_54_-Flag fragment (Fig. 4a). To assess the capacity of *P. aeruginosa* T3SS to inject transcription factors into ES cells, plasmids pExoS_54_F-Gata4, pExoS_54_F-Mef2c and pExoS_54_F-Tbx5 were each electroporated into three *P. aeruginosa* strains, Δ*exsA*, Δ*popD* and Δ8, respectively. The resulting transformants were cultured in L-broth in the presence of 5 mM EGTA for 3 hours to induce the type III secretion. Culture supernatants and cells pellets were separated by centrifugation and then subjected to Western blot analysis using anti-Flag antibody. The strain Δ*exsA* is deleted of a transcriptional activator for the T3SS regulon, thus defective of the type III secretion^36^. Strain Δ*popD* contains a functional T3SS that is capable of protein secretion into culture medium, but it is defective in protein injection into the host cells due to the lack of PopD protein required for the formation of the pore on host membrane through which the needle injects effectors^37^. Figure 4b shows that none of the fusion proteins were expressed or secreted by the T3SS-defective mutant Δ*exsA*, however, both Δ*popD* and Δ8 strains were capable of producing and secreting the fusion proteins, indicating that the ExoS_54_F-TF fusions could be produced and secreted into culture medium in a T3SS-dependent manner.

**Figure 5:**
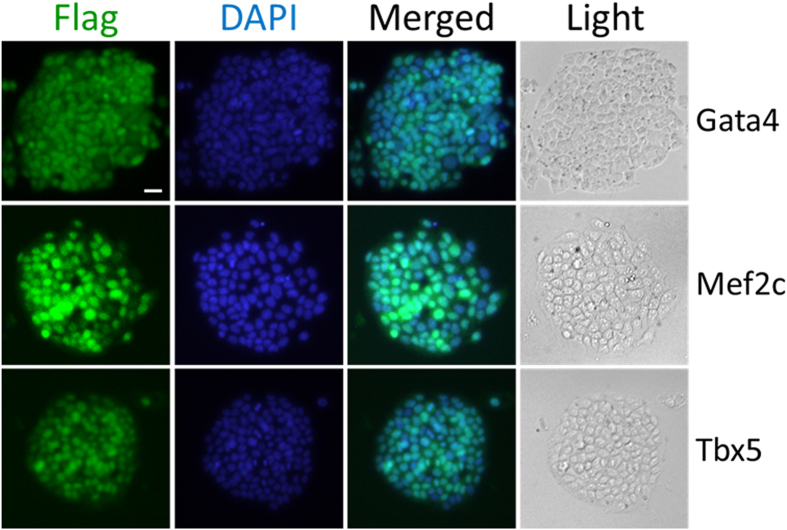
TF delivery into mESCs. mESCs were infected with Δ8/Gata4, Δ8/Mef2c, Δ8/Tbx5 respectively, for 3 hours at MOI 50 and subsequently fixed and immunostained with anti-Flag to illuminate translocated ExoS_54_-Flag-TF proteins. ; Nuclei were stained with DAPI. Bar is 100 μM.

To test delivery of the transcription factors into ESCs, strains of Δ8/Gata4, Δ8/Mef2c or Δ8/Tbx5 were individually co-incubated with mouse ES cells at MOI of 50 for 3 hours. Free floating bacterial cells were subsequently removed by successive washes with PBS (see Supplementary Fig. 3), then the ESCs were examined for intracellular fusion proteins by immunoblot or directly immunofluorescence staining. As the results shown in Fig. 4c, none of the fusion factors were injected into ESCs by Δ*exsA* or Δ*popD*, although the fusion proteins were made by the Δ*popD* strain. In contrast, all of the fusion proteins could be injected into ESCs by strain Δ8, indicating that the injection of the fusion proteins occurs in a T3SS-dependent manner (Fig. 4d). In addition, the injections occurred in a dose-dependent manner, as there were more translocated fusion proteins when the MOI increased from 50 to 100 (Fig. 4c). Most importantly, all three transcription factors (GMT) could be detected in the nucleus of ESCs (Fig. 5). The injection seems to occur uniformly on ES cell population, reaching almost 100% target cells at MOI of 50 within 3 hours of infection time. These results demonstrated that the GMTs can be effectively delivered into ES cells by the bacterial T3SS-based protein delivery tool and the translocated proteins are effectively targeted to the nucleus.

### Subcellular localization and half-lives of the T3SS-injected TFs

**Figure 6:**
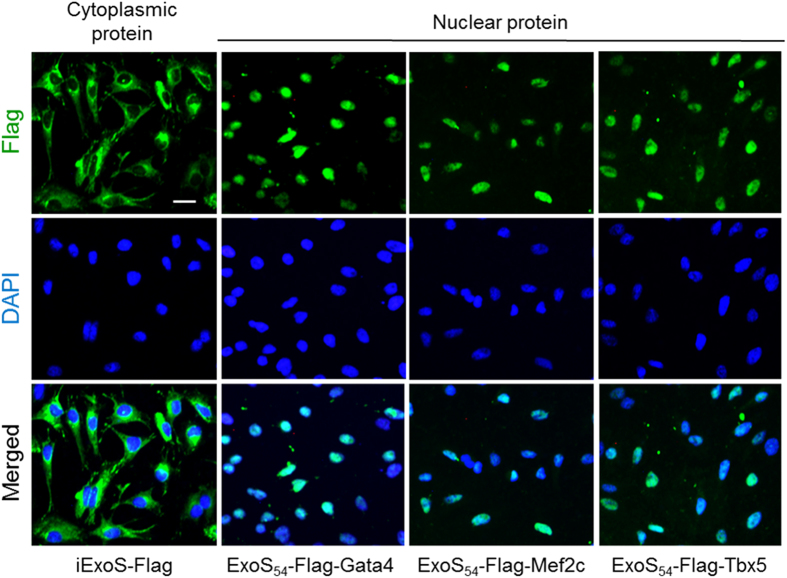
Subcellular localization of injected TFs. Immunohistochemistry of HeLa cells following infection by ∆8/piExoS-Flag, ∆8/pExoS_54_F-Gata4, ∆8/pExoS_54_F-Mef2c or ∆8/pExoS_54_F-Tbx5 (3-4 h at MOI 50). Cells were stained with anti-Flag antibody; nuclei were stained with DAPI. Bar = 50 μm.

For transcription factor delivery, a key question is whether the ExoS_54_-TF fusions can correctly be targeted into the nucleus of eukaryotic cells where they are expected to function by binding to specific DNA sequences. Since mESCs have a large nucleus with minimal cytoplasm, and individual cells are clustered together to form colonies, ESC is not an ideal cell type for the study of protein subcellular localization. Here, HeLa cell line was used to study nucleus targeting of injected proteins. Strain Δ8 carrying iExoS-Flag, or the ExoS_54_-Flag fused with Gata4, Mef2c or Tbx5 were used to infect HeLa cells at MOI of 50 for 3-4 hours. The intracellular distribution of the translocated proteins was monitored by immunofluorescence staining with an anti-Flag antibody. As Fig. 6 shows, all three ExoS_54_-TF fusions were predominantly delivered to the nucleus within 4 hours, whereas iExoS-Flag is exclusively found in the cytoplasm, indicating that the N-terminal ExoS_54_-Flag fragment does not interfere with the nuclear localization of the fused transcriptional factors.

**Figure 7:**
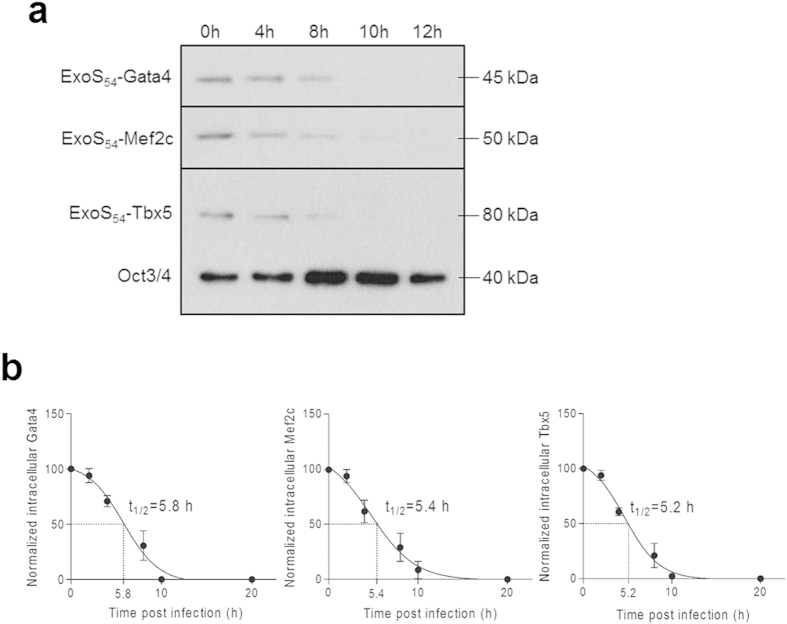
Intracellular stability of injected proteins. mES cells were infected with ∆8/pExoS54F-Gata4, ∆8/pExoS_54_F-Mef2c and ∆8/pExoS_54_F-Tbx5 at MOI of 50 for 3 hours, respectively. (**a**) Post the bacterial infection (time 0 h), nuclear proteins were extracted at the indicated time and subjected Western blot. Anti-Flag antibody was used to detect the injected TF fusion, anti-Oct3/4 antibody was used to detect endogenous TF Oct3/4. (**b**) Quantification of Western blots by Image J, half-life (t_1/2_) was determined by time vs. injected protein curve.

Intracellular proteins are constantly subjected to degradation by proteases at various rates, which was shown dependent on the exposed N-terminal residues in both prokaryotes and eukaryotes^38^. Half-lives of the three ExoS_54_-TFs fusion proteins within ESCs were determined by Western blot analysis, using the endogenous transcription factor Oct3/4, an undifferentiated ESCs marker, as an internal control. As shown in Fig. 7a, the injected proteins were gradually degraded in a time-dependent manner, till 10 hours post infection. Quantification of the protein band intensities indicated that the half-lives of three ExoS_54_-TF fusions were all around 5.5 hours (Fig. 7b).

### GMT delivery promotes *de novo* differentiation of ES cells toward cardiomyocytes

**Figure 8:**
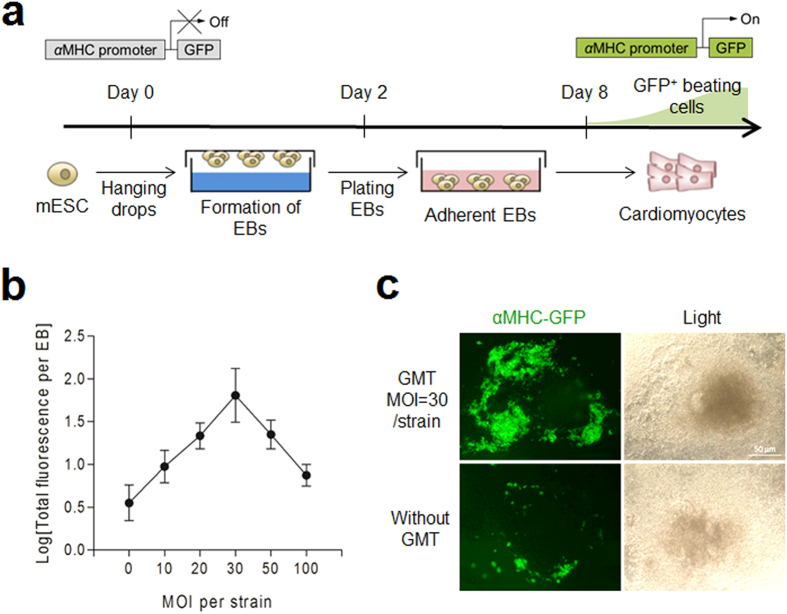
GMT delivery promotes *de novo* differentiation of ESC-CMs. (**a**) Protocol for differentiation of cardiomyocytes from embryoid bodies (EBs). mESCs were dissociated into single cells on day-0 and cultured in suspension for 2 days in hanging drops and then plated on gelatin coated culture plate. (**b**) GMT injection at various MOI on day-5, and total GFP fluorescence of each EB were measured on day-12. (**c**) Live cell images showing αMHC-GFP^+^ cardiomyocytes in 12-day old EBs.

Mouse ES cell line αMHC-GFP, with a GFP transgene driven by α-cardiac myosin heavy chain promoter which is active only in cardiomyocytes^39^, was cultured in hanging drops for 24 hours to form embryoid bodies (EBs). The EBs were transferred into 24-well tissue culture plates on day 2, and allowed for spontaneous differentiation. Starting from day-8, ESC-derived cardiomyocytes (ESC-CMs) can be detected by αMHC-GFP^+^ fluorescence and even spontaneously beating clusters (Fig. 8a). The EBs were subjected to GMT injection individually or in combination on EB day-4. After 12 days of differentiation, EBs injected of the GMT together showed significantly higher GFP fluorescence intensity compared to those injected of the individual factors (see Supplementary Fig. 4a). Also, a combined delivery of the GMT on day-5 resulted in the highest expression levels of cardiomyocyte marker genes *Nkx2.5* and *αMHC*, indicating the most effective promotion of cardiac program (see Supplementary Fig. 4b). Bacterial delivery of GMT with a total MOI of 150 did not lead to morphological change of the EBs during differentiation compared to EBs without bacterial infection (see Supplementary Fig. 4c). To determine the optimal MOI, EBs were infected by each transcription factor delivery strain at MOIs of 10, 20, 30, 50 and 100 on day-5 and the total GFP fluorescence per EB (TF/EB) was recorded on day-12. As the results shown in Fig. 8b, MOI of 30 for each strain, thus the EBs infected by all three delivery strains (GMT) had an overall MOI of 90, showed the highest efficiency of CMs differentiation. Compared to the spontaneously differentiated EBs, more GFP^+^ cardiomyocyte-like cells (αMHC-GFP) appeared in EBs that were injected of GMT combination on day-5 (Fig. 8c). These results demonstrated that GMT combination was able to promote ESCs differentiation into cardiomyocyte-like cells (αMHC-GFP^+^) with a nonlinear (bell curve) dose-dependent manner, indicating that proper ratio and stoichiometry of each transcription factor were necessary for high efficiency differentiation.

### Determination of an optimal ratios of the three transcriptional factors for cardiomyocyte differentiation

Response surface methodology (RSM) is a collection of statistical and mathematical techniques used to improve and optimize complex processes^40^,^41^. The main advantage of RSM is its ability to reduce the number of experimental trials needed to evaluate multiple parameters and their interactions, in order to provide sufficient information for statistically acceptable results^42^,^43^. In this study, the Box-Behnken design of RSM was chosen to optimize the relative ratio of three factors (Gata4, Mef2c and Tbx5). The experimental design and the corresponding responses were presented in Supplementary Table 1. The statistical significances of the model and each coefficient were checked by ANOVA analysis and the results are presented in Supplementary Table 2. The relationship between response (*Y*) of total fluorescence intensity per EB (TF/EB) and a number of variables denoted by *X*_1_, *X*_2_, *X*_3_, *X*_1_X_2_, *X*_1_X_3_, *X*_2_X_3_, *X*_1_^2^, *X*_2_^2^ and *X*_3_^2^ (*X*_1_, *X*_2_ and *X*_3_ represent MOIs of Gata4, Mef2c and Tbx5 delivery bacterial strains, respectively) could be approximated by a second-degree model. ANOVA analysis showed that the second-degree model was significant (*P* < 0.01). Among the variations, only *X*_1_, *X*_2_, *X*_3_, *X*_1_^2^ and *X*_3_^2^ had significant effect on the model, with *P*-values less than 0.05 (Table S2). Thus, the experimental results could be modeled by a second-order polynomial equation to explain the dependence of total GFP fluorescence intensity of each EB (*Y*) on the different factors:


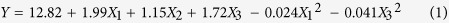


**Figure 9:**
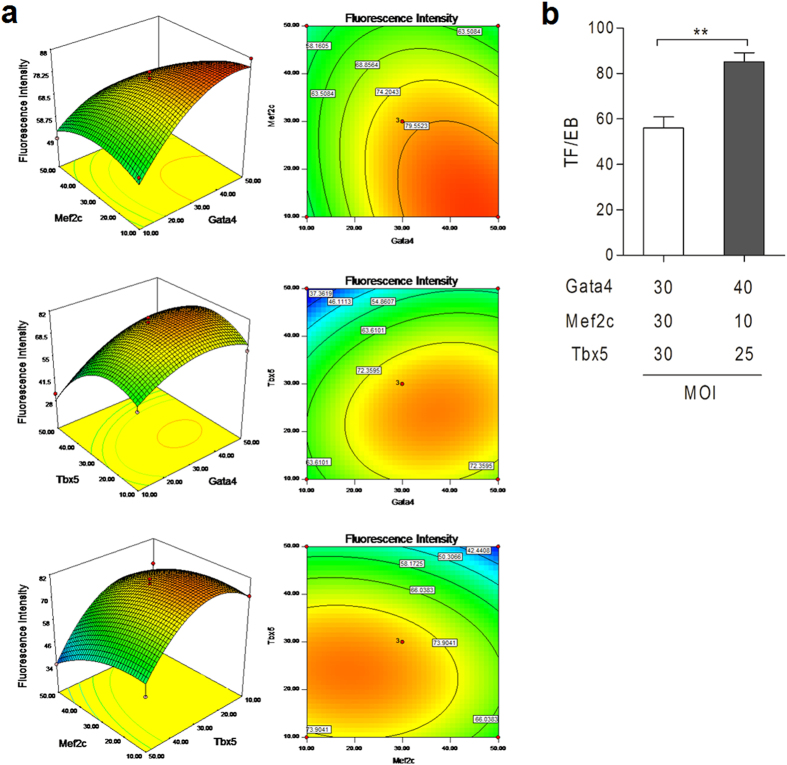
Determination of an optimal ratios of GMT for cardiomyocyte differentiation. (**a**) Response surface plots showing effects of various parameters on fluorescence intensity of EBs and contour plots showing predicted optimal response. (**b**) Total fluorescence per EB (TF/EB) following injection of GMT at the relative ratios before and after optimization.

The fit of the model was evaluated by determining coefficient *R*^2^. The regression equations obtained showed an *R*^2^ value of 0.9492, indicating that the model could explain 94.92% of the variability in the response. The response surface plots and their respective contour plots for the predicted response *Y* based on the second-order model are shown in Fig. 9a. They provided prediction of the optimal MOI values for Gata4 (*X*_1_), Mef2c (*X*_2_) and Tbx5 (*X*_3_) to be 40, 10, and 25, respectively. The corresponding experiments were carried out to compare the average *Y* values between MOI = 30:30:30 and MOI = 40:10:25 for the three strains (Δ8/Gata4, Δ8/Mef2c and Δ8/Tbx5). The average fluorescence intensity of EBs was indeed significantly higher with the delivery of the three factors at the optimal ratio (Fig. 9b).

### Multiple rounds of GMT delivery improve ESC-CMs differentiation

**Figure 10:**
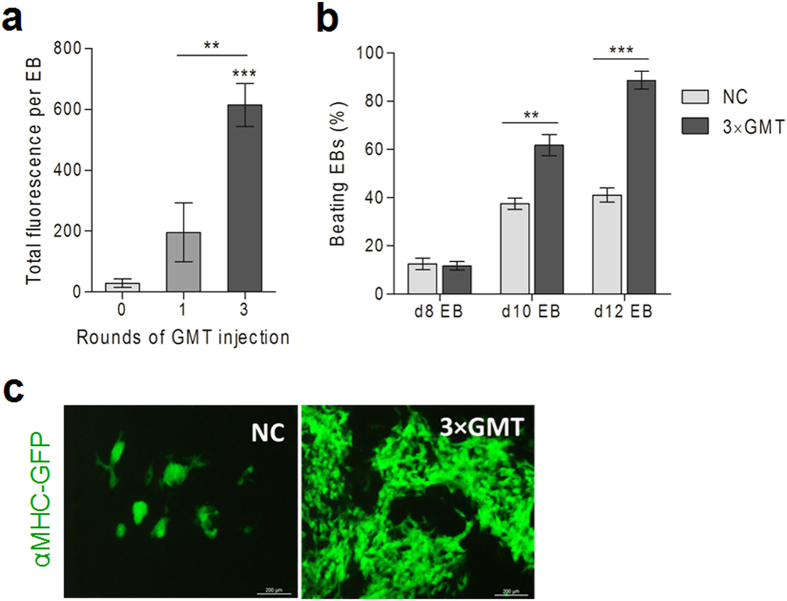
Multiple rounds of GMT delivery improves ESC-CMs differentiation. (**a**) GMT injection at MOI = 40G:10M:25T for one time on day-5 or 3 times on days-5, 7 & 9, then TF/EB were recorded on day-12. Data represents mean ± SD, (n > 20); **0.01 < *P* < 0.05, ***0.001 < *P* < 0.01. (**b**) Percentage of EBs containing beating areas. More than 40 embryoid bodies were counted per condition per day (48 EBs per condition in total). NC, negative control (EBs without any treatment). (**c**) Live cell images showing GFP^+^ contraction cluster of 12-day old EBs.

**Figure 11:**
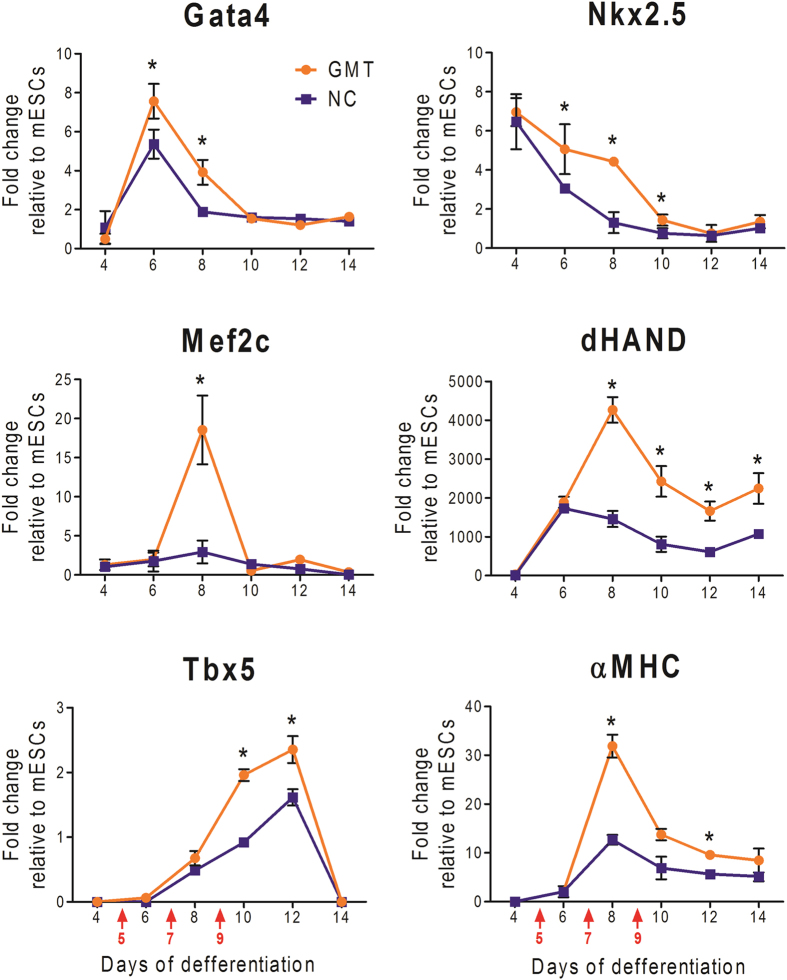
Relative expression levels of cardiac marker genes. EBs with three rounds of GMT delivery (GMT) or non GMT-treated control (NC) were subjected to quantitative PCR analysis and normalized to the mES cells. Endogenous GMT, cardiac mesodermal markers NKX2.5 and dHAND, and cardiomyocyte marker MYH6. Red arrows indicate the days of GMT delivery. Error bars represent SEM of 3 biological replicates. *P < 0.05.

As the half-lives of ExoS_54_-TFs were only 5.5 h, we predicted that multiple rounds of GMT delivery may enhance their influence on ESC-CMs differentiation. Effects of one time injection of GMT on day-5 was compared to that of multiple rounds of injection at the optimal MOI ratio of the three factors (GMT), evaluating the GFP fluorescence intensity of each EB on day-12. Multiple rounds of GMT delivery (on days 5, 7 and 9) dramatically increased the fluorescence intensity of EBs compared to the one time GMT delivery group, while the latter group was significantly higher than the control group without GMT delivery (Fig. 10a). We also found a continued increase in the number of beating EBs in 3 rounds of GMT delivery (3 × GMT) group, large contractile areas appeared in ~85% of the 3 × GMT treated EBs on day-12, while only 40% spontaneous differentiated EBs had beating areas that are much smaller in sizes (Fig. 10b). Representative beating clusters composed of GFP^+^ cells are shown in Fig. 10c (see Supplementary Video 1,2,3). Reverse transcription quantitative polymerase chain reaction analysis was performed to further evaluate the effect of exogenous GMT protein delivery on the expression levels of selected cardiac gene. GMT proteins were delivered into EBs at three time points (day-5, 7, and 9) while cardiac gene expression was determined at six time points (day-4, 6, 8, 10, 12, and 14), using EBs without GMT delivery as control. Cardiac transcription factor Gata4, Mef2c, Tbx5, Nkx2.5 and dHand, known as early cardiac progenitor markers, as well as the cardiomyocyte structural gene *MYH6* (αMHC) were increased dramatically by 3 rounds of GMT delivery (Fig. 11). These results demonstrated that multiple rounds of GMT delivery significantly improve the efficiency of EB differentiation into cardiomyocytes.

### Activin A shows an additive effect on the ESC-CMs differentiation promoted by the GMT injection

**Figure 12:**
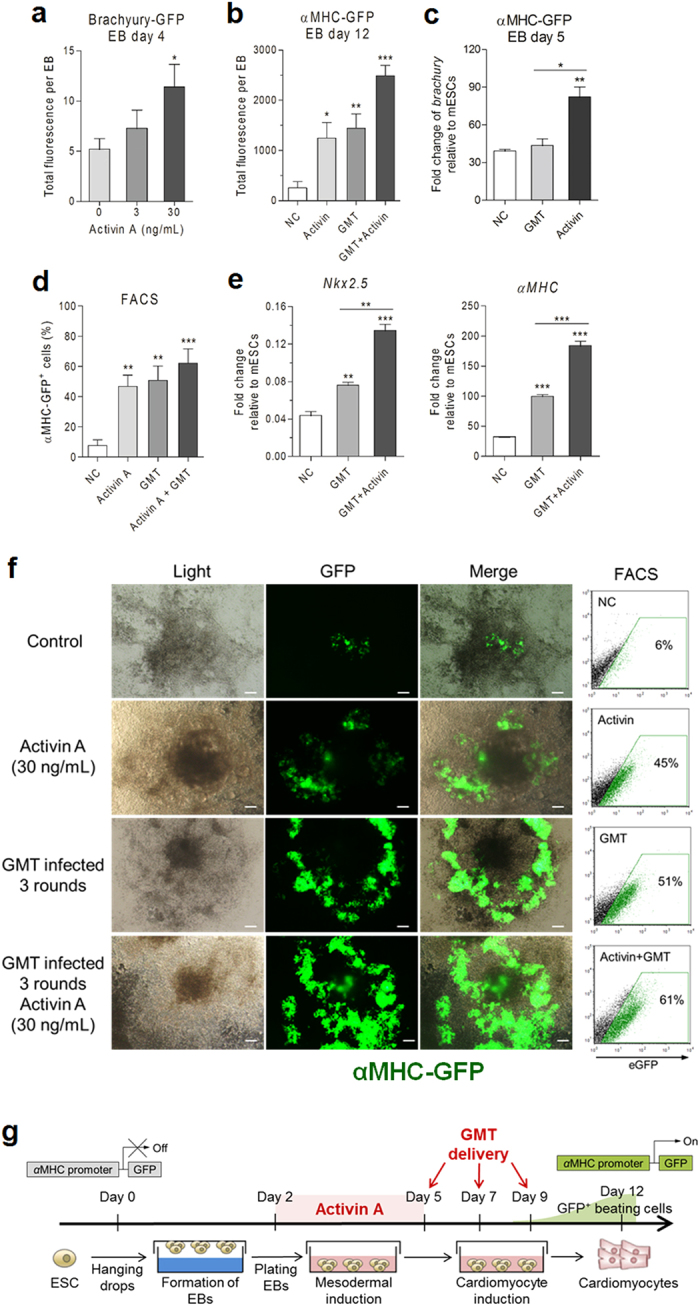
Additive effect of Activin A on the ESC-CMs differentiation promoted by the GMT deliveries. (**a**) Activin A treatment on day-2, and GFP fluorescence intensity measurements of mesodermal marker Brachyury-GFP on day-4. (**b**) Fluorescence intensities of EBs with or without GMT injection in the presence or absence of Activin A (30 ng/mL) in culture medium. (**c**) GMT combination was delivered on EB day-4. Quantitative PCR measurements of Brachury on EB day-5. (**d**) Fluorescence-activated cell sorting (FACS) analysis of αMHC-GFP positive cells in 12 day-old EBs. (**e**) Quantitative PCR measurements of Nkx2.5 and αMHC in EB on day-12. NC, negative control; GMT, 3 rounds of GMT delivery; GMT^+^ Activin, 3 rounds of GMT delivery plus 30 ng/mL Activin A pre-treated for 3 days. Data represents mean ± SD, (n > 3); *P < 0.05, **0.001 < *P* < 0.01, *** *P* < 0.001. (**f**) Live cell images showing αMHC-GFP^+^ cardiomyocytes of 12-day old EBs. Controls were spontaneously differentiated EBs. Representative FACS analysis and the percentage of ESC derived αMHC-GFP^+^ cardiomyocytes. (**g**) Protocol for differentiation of cardiomyocytes in EB system with Activin A treatment and GMT delivery.

Embryoid bodies (EBs) are three-dimensional aggregates of pluripotent stem cells. ESCs within EBs undergo differentiation and cell specification along the three germ lineages—endoderm, ectoderm, and mesoderm—which comprise all somatic cell types^44^. The cardiac lineages develop from subpopulations of the mesoderm induced in a defined temporal pattern, and expression of Brachyury is commonly used to monitor the onset of mesoderm induction in the ESCs differentiation studies^45^. It had been reported that treatment with proper stoichiometry of Activin A induces mesodermal fate from both mouse and human pluripotent stem cells (PSCs), where high levels of Activin A promote definitive endoderm, moderate levels promote cardiac mesoderm, and low levels promote mesoderm of vascular and hematopoietic lineages^16^,^46^,^47^. To determine the optimal amount of Activin A required for mesoderm differentiation, we generated EBs using a mouse ESC line with Brachyury-GFP reporter gene and treated with various concentrations of Activin A from day-2 to day-4. As shown in Fig. 12a, Bry-GFP^+^ cells increased in a dose-dependent fashion, with more than two folds increase in GFP fluorescence intensity by day-4 following stimulation with 30 ng/mL of Activin A. For the CMs differentiation from αMHC-GFP ESCs, addition of 30 ng/mL of Activin A from day-2 to day-5 resulted in about 5-fold increase of GFP fluorescence intensity in EBs (Fig. 12b). To further determine whether Activin A could directly induce mesodermal formation, expression of early mesodermal marker Brachyury was determined by q-PCR. On day-5 of EB differentiation, Activin A treated EBs showed higher expression level of the Brachyury, while GMT delivery did not result in such an up-regulation (Fig. 12c), indicating that GMT exert their regulatory effect after the mesodermal stage, while Activin A promotes ESC differentiation towards mesodermal cells.

Interestingly, when we combined Activin A and GMT treatments, the fluorescence intensity of EBs on day-12 increased by about 10 folds compared to those untreated EBs (Fig. 12b). From morphology and fluorescent-assisted cell sorting (FACS) assays (Fig. 12f), about 6% of the αMHC-GFP^+^ cells appeared in the spontaneously differentiated EBs (control), while treating with Activin A for 3 days resulted in 45% of MHC-GFP^+^ cells appeared in or around the center of EBs. Three rounds of GMT deliveries resulted in 51% of MHC-GFP^+^ cells, with the GFP^+^ cells mostly located outside of the EB centers. Most strikingly, combination of the Activin A and GMT deliveries resulted in 61% MHC-GFP^+^ cells in the whole EB cells, representing a 10-fold higher efficiency comparing to the spontaneous differentiation (Fig. 12d), with the GFP^+^ cells appearing both inside and outside of the EB centers (Fig. 12f). In addition, by day-12 of the differentiation, the expression levels of cardiac markers gene Nkx2.5 and α-MHC were significantly higher in the Activin A plus GMT delivery group, compared to either GMT delivery alone or negative control group (Fig. 12e). These results clearly demonstrated an additive effect of the T3SS-mediated GMT delivery and Activin A treatment on the ESC-CMs formation. As summarized in Fig. 12g, the optimal protocol was determined as 30 ng/mL Activin A treatment from day-2 to day-5, followed by T3SS-mediated GMT delivery at MOIs of 40G:10M:25T for 3 times on days 5, 7 and 9, then assessing the differentiation on day-12 and beyond.

### Characterization of ESC-CMs

**Figure 13:**
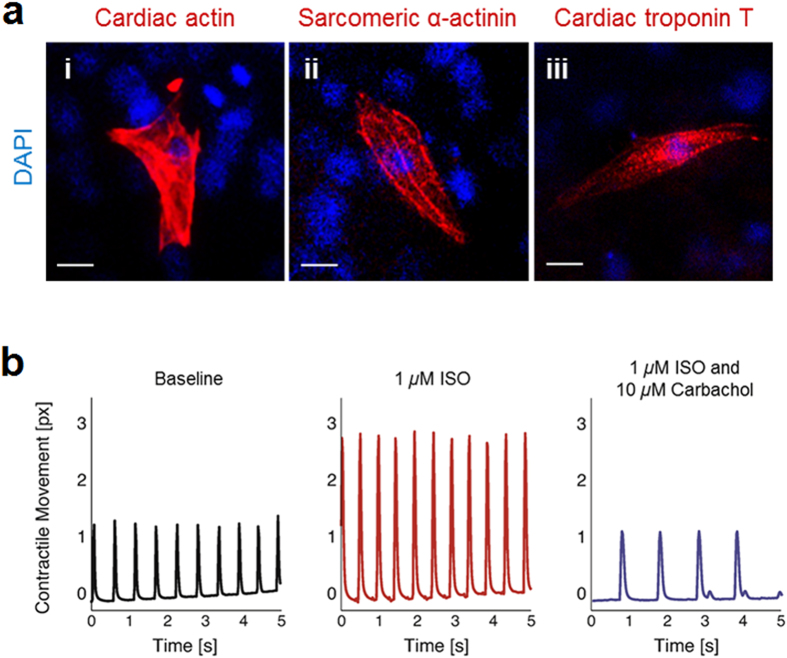
Characterizations of GMT induced ESC-CMs. (**a**) Single cells dissociated from day-12 EBs were stained with anti-cardiac actin (i), anti-sarcomeric α-actinin (ii) and anti-cardiac troponin T (iii). Nuclei are stained with DAPI (blue). (**b**) Contractile movement analysis demonstrating functional expression and integration of β-adrenergic and muscarinic signaling in ESCs-derived cardiomyocytes with rhythmic contractile movement. The magnitude and frequency of contraction increased after administration of the β-adrenergic agonist isoproterenol (ISO). Subsequnt application of carbachol led to a blockage of the ISO effect.

To further evaluate the ESC-CMs, presence of sarcomeric proteins were detected by immunofluorescence analyses. Cells from 12-day differentiation protocol (Activin A plus GMT delivery) was trypsinized and replated. ESC-CMs were revealed of well-organized cross-striation and positive for cardiac α-actin, sarcomeric α-actinin, and cardiac troponin T (Fig. 13a), demonstrating that ESC-CMs express the cardiac isoform of marker proteins.

One of the most critical determinants of normal cardiac physiology is the intact response to hormones and transmitters of the central nervous system^48^,^49^. Accordingly, we studied effects of β-adrenergic stimulator isoproterenol (ISO, 1 μmol/L) and cholinergic agonist carbachol (10 μmol/L) on contractile movement of 12-day old EBs. Videos of ESC-CMs were recorded at 50 fps, and a cross-correlation algorithm was used to detect pixel movement. Average pixel movement over the entire image is plotted versus time. As shown in Fig. 13b, application of ISO led to a typical and comparable increase of the contraction frequency and magnitude of movement (Fig. 13b, middle panel) compared with basal frequency (left panel). Subsequent application of carbachol effectively blocked the ISO effect on the beating cells by slowing their contraction frequency as well as magnitude (right panel), indicating the presence of intact and coupled β-adrenergic as well as muscarinic signaling cascades.

## Discussion

**Figure 14:**
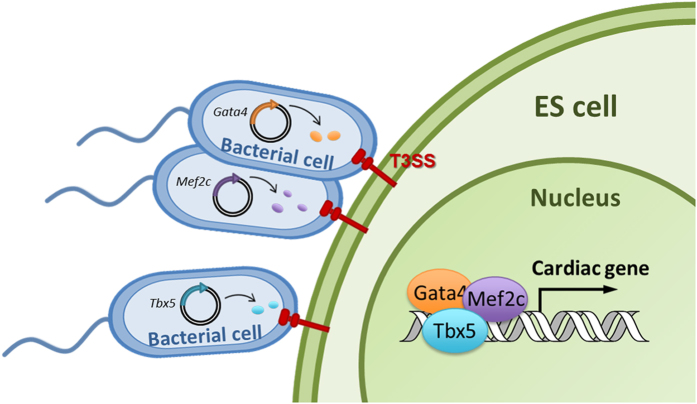
Schematic representation of directed differentiation of ES cells into CMs by bacterial injection of transcription factors.

In this study, we have developed a bacterial T3SS-based transcriptional factor (TF) delivery tool that could be used in directing ES cell differentiation *in vitro*. By cloning into the expression vector pExoS_54_F, three TF genes (GMT) were fused in-frame with the T3SS secretion signal ExoS_54_ sequence. Upon physical contact between bacteria and ES cells, a condition known to activate T3SS, the ExoS_54_-TF fusions are expressed inside bacterial cells followed by a rapid injection into ESCs via the T3SS apparatus (Fig. 14). In eukaryotic cells, transcription factors contain nuclear localization signals (NLS), consisting of one or more short sequences of positively charged lysines or arginines exposed on the protein surface, which direct them into the nucleus^50^. However, the rates at which various TFs target into nucleus vary, in some cases they are regulated by posttranslational modifications^51^,^52^. Among the GMT, Mef2c seems most rapidly targeted into nucleus, whereas Gata4 and Tbx5 were slower and observed in both the cytosol and nucleus by 3 hours of infection, however, by 4 hours of the infection time, most of injected proteins were localized into the nucleus (see Supplementary Fig. 5). Depending on the TFs, fusing additional NLS at N- or C-terminus may alter the rate and efficiency of nuclear targeting process. In our recent studies, a pair of large TALEN proteins were injected into mammalian cells via T3SS by fusing to the ExoS_54_-secretion signal where the translocated TALEN proteins were efficiently targeted into nucleus by a NLS from SV40 T-Antigen^10^.

As we have shown in this study, the bacterial delivery strains can completely be eliminated by a simple antibiotic treatment, leaving no viable bacterial cells behind after 12 hours (Fig. 3b). Furthermore, as demonstrated in out earlier studies, multiple rounds of bacterial infection did not alter the characteristics of the target cell types, especially the pluripotency of stem cells, maintaining their self-renewal capacity^9^.

The injected TFs are gradually degraded in a time-dependent manner, till 10 hours post infection, which is similar to other proteins that we had previously delivered into the mammalian cells^10^. The N-terminal amino acids are known to influence the half-lives of proteins, where proteins with segments rich in proline, glutamic acid, serine, and threonine have shorter half-lives^38^,^53^. In this study, the N-terminal 54 amino acids of ExoS was fused to all the three TFs, thus the N-terminal features are uniform for the GMT, accordingly, the ExoS_54_-TFs had similar half-lives of approximately 5 h. However, T3SS-delivered ExoS are known to localize to cytosol and enriched on perinuclear region, with a short half-life of only 20 min^54^. Such drastic difference in intracellular half-lives might be due to a lower abundance of proteases within nucleus and/or there are protective mechanisms that prevent mammalian transcriptional factors from proteolytic degradation. There are many factors shown to affect protein degradation, including deamination of glutamine and asparagine, oxidation of cystein, histidine, and methionine, presence or absence of stabilizing ligands, protein modifications by carbohydrate or phosphate groups, presence of free α-amino group or negative charges, and the structural flexibility of the protein^38^. Therefore, depending on the experimental needs, the intracellular stability of TFs can be controlled by engineering specific amino acid sequence under the premise of maintaining their normal activity.

Here, we have developed a novel protein delivery tool based on the T3SS of *P. aeruginosa*. Through systemic dulling of the bacterial cytotoxicity, this protein delivery tool was applied to stem cells. By fusing with a Type III secretion sequence, transcriptional factors have been directly injected into the ES cells, avoiding the introduction of foreign genetic materials (DNA/RNA). Also, due to the simplicity of manipulation and short half-lives of the injected proteins, transcriptional factors can be delivered at anytime, in any order, for desired period of time and amounts. Using this method, we have demonstrated that three transcriptional factors (GMT) relevant to early heart development can promote ESC-CMs differentiation. The exogenous GMT protein deliveries activated global cardiac genes expression, promoting cellular differentiation towards cardiomyocytes. In addition, the optimal ratio of GMT was carefully determined by RSM, adjusting the injection dose of each TF by changing its MOI. GMT injection plus pre-treatment with mesodermal inducer Activin A have shown an additive effect on promoting ESC-CMs differentiation, with the resulting CMs expressing cardiomyocyte-specific myosin and displayed well-organized cross-striations. The ESC-CMs also showed spontaneous rhythmic contractile movement and normal hormonal response, demonstrating that functional cardiomyocytes can be generated by the injection of defined transcriptional factors.

Cardiac development is a dynamic process that is tightly orchestrated by the sequential expression of multiple signal transduction proteins and transcription factors working in a combinatory manner^55^. Three main steps are required to generate cardiomyocytes from pluripotent stem cells: (i) mesoderm induction and patterning, (ii) cardiac specification, and (iii) cardiomyocyte maturation^55^. The first step (i.e., mesoderm induction) in cardiac differentiation from pluripotent stem cells has been well characterized. Numerous studies have demonstrated that Wnts, BMPs, and transforming growth factor (TGF) β-family member Nodal efficiently induce mesoderm^56^,^57^. Interestingly, Activin A, a mimic of Nodal, has been shown to be critical for Brachyury^+^ mesendoderm lineages in a concentration-dependent manner^47^. In this study, Activin A showed an additive effect on the ESC-CMs differentiation promoted by the GMT injection, while GMT delivery alone could not increase the expression level of mesodermal maker Brachyury, indicating that GMT mainly affect the phases of cardiac specification and cardiomyocyte maturation, beyond the mesodermal differentiation.

Recent protocols of cardiomyocyte differentiation have largely replaced undefined serum with the sequential application of growth factors and, more recently, small molecules^17^,^55^. Many of the growth factors are costly to obtain, degrade rapidly, and do not readily diffuse into complex multicellular aggregates. In addition, they exhibit lot-to-lot variation in their bioactivity. For some applications, large-scale production of CMs from PSCs is of critical importance. Clinical therapies envisioning the use of hPSC-derived CMs may require 10^8^ to 10^9^ cells per patient dose, which reflects the amount of working myocardium lost in a large myocardial infarction^16^,^58^,^59^. Additionally, high-throughput screening of libraries of small molecules using PSC-derived CMs for safety or efficacy signals will require large numbers of CMs^16^. To enable such applications, the differentiation process will need to be scalable. Since no protein or DNA/RNA purification step is required, adaptation of the bacterial T3SS-based TF delivery system to a large production scale simply involves scaling up of the culture of bacterial delivery strains. The ease of growing bacterial cultures makes the process amendable to scale up production.

Forced expression of cell type specific transcriptional factors became a powerful approach to reprogram cells. However, most cellular differentiation processes go through multiple developmental stages, each requiring specific sets of transcriptional factors, thus often not successful by simple forced constitutive expression of defined transcriptional factors. Even with a clear understanding of the natural developmental processes, we are unable to mimic the natural developmental processes *in vitro* using currently available technologies, mainly due to the transient nature of transcriptional activation in specific orders. The T3SS mediated delivery of transcriptional factors in the form of protein can overcome this difficulty by controlled delivery of multiple proteins into a large number of target cells, enabling us to closely mimic the natural transient waves of transcriptional activation, thus likely to succeed in reprograming pluripotent stem cells into any desired lineages. Our future goals will revolve around the experimental demonstration of such powerfulness of our novel protein delivery tool.

## Materials and Methods

### Bacterial Strains

The bacterial strains and plasmids used in this study are listed in Table 1. *P. aeruginosa* were grown in Luria (L) broth or on L agar plates at 37 °C. Antibiotics were used at a final concentration of 150 mg carbenicillin per mL.

### Cell Culture

Human HeLa cell line (cervical cancer) was purchased from the American Tissue Culture Collection (ATCC). HeLa cells were cultured in Dulbecco’s Modified Eagle Media (DMEM) supplemented with 10% FBS and 1% penicillin/streptomycin (Gibco). Cells were incubated at 37 °C with 5% CO_2_. Mouse ES cell line R1 was kindly provided by Dr. Andras Nagy, Toronto, Ontario, Canada; mouse ES cell line CGR8 with an EGFP transgene targeted to the α-cardiac myosin heavy chain promoter (MHC-GFP)^39^ was kindly provided by Dr Richard Lee, Harvard University, Boston; mouse ES cell line 129/Ola with an EGFP transgene targeted to the Brachyury locus (Brachyury-GFP)^60^ was kindly provided by Dr Gordon Keller, Mount Sinai School of Medicine, New York, NY. Mouse ESCs were routinely cultured and expanded in LIF medium on 0.1% gelatin (Millipore) coated tissue culture plates. The LIF medium was composed of KnockOut Dulbecco’s modified Eagle’s medium (DMEM; Gibco) supplemented with 10% knockout serum replacer (SR, Gibco), 1% fetal bovine serum (FBS, Gibco), 25 mM Hepes, 300 μM monothioglycerol (Sigma), 1% penicillin-streptomycin and 1 mM L-glutamine (Gibco), and 10^3^ units/mL recombinant mouse leukemia inhibitory factor (LIF, Millipore). Ciprofloxacin was added at final concentrations of 20 μg/mL, where noted to clear the protein delivery strain of *P. aeruginosa*.

### Cytotoxicity Assays

Cells were infected by *P. aeruginosa* for different hours as described above. After infection, the cells were washed and incubated with 0.25% Trypsin for 5 minutes. The number of cells were then counted under microscope. The lactate dehydrogenase (LDH) release assay used CytoTox96 (Promega) and followed the manufacturer’s instruction.

### Protein Production and Secretion Assay

*P. aeruginosa* strains were grown overnight in 2.0 ml of Luria broth containing carbenicillin (150 μg/ml) at 37 °C. Overnight cultures were then inoculated at 5% into fresh L broth plus antibiotics, where 5 mM EGTA and 0.2% serum were supplemented for type III inducing condition. *P. aeruginosa* strains were grown in a shaking incubator at 37 °C for 3–5 h, after which bacterial cells were centrifuged at 20,000 g for 2 min. Bacterial supernatants were collected, precipitated with 15% trichloroacetic acid (TCA, 20 × concentration), resuspended in 1 × SDS protein sample buffer and boiled for 15 min before Western Blot analysis.

### Protein Injection Assay

ES cells were seeded at approximately 70% confluence in antibiotic-free medium. *P. aeruginosa* strains were grown at 37 °C in Luria broth containing carbenicillin until reaching an optical density (OD_600_) of 0.8. ES cells were co-cultured with bacteria at a multiplicity of infection (MOI) of 100 for 3 hours. Infection was terminated by washing cells three times with PBS and growing the cells on ES medium containing 20 μg/mL ciprofloxacin. In the case of immunofluorescence analysis (see below), infections were stopped by fixation with paraformaldehyde (PFA).

For Western Blot analysis, cells were infected as described above. Immediately following infection, cells were washed, collected by digestion with 0.25% trypsin, and centrifuged at 500 × g for 10 min. The cell pellets were lysed in sodium dodecyl sulfate polyacrylamide gel electrophoresis (SDS-PAGE) loading buffer, and boiled for 15 min.

### Western Blotting

Western blot assays were performed as previously described^61^. Secretion and injection samples were separated on 4–20% gradient SDS-PAGE gels (Bio-Rad). Proteins were transferred onto PVDF membranes and subjected to immunoblotting using an anti-Flag antibody (mouse M2 monoclonal Ab; Sigma) for GMT and anti-β-actin (Santa Cruz) for actin, with 1000-fold dilutions.

### Cardiac differentiation of ES cells

All murine ESC lines were differentiated as previously described^62^. To initiate embryoid body (EB) formation, “hanging drops” composed of 2000 cells in 30 μL of differentiation medium were generated (day-0 of differentiation). The differentiation medium was based on Iscove’s modified Dulbecco’s medium (IMDM, Gibco) and supplemented with 20% heat inactivated FBS, 0.5 mM monothioglycerol, lacking supplemental LIF. On day-2 of differentiation, the EBs were transferred into gelatin coated 24-well plates with 1–2 EBs per well and cultivated for 2 additional days. From day 5 until day 12, differentiation medium was replaced every 2–3 days. Images of EBs were captured at×5 magnification with a Leica DMIRB inverted phase contrast fluorescence microscope with a DFC425 camera (Leica) and processed using the Leica Application Suite (LAS) microscope software. The microscopic images of the fluorescent EBs were further analyzed for the quantitative analysis of total fluorescence in each EB. Total fluorescence per EB (TF/EB) was calculated in an excel sheet by applying the measurements obtained from the EBs using Image J software. Total fluorescence per EB (TF/EB) = Integrated Density - (Area of selected EB × Mean fluorescence of background readings).

### Flow cytometry

For fluorescence-activated cell sorting (FACS) analysis, single cells were dissociated from embryoid bodies on day-12 using TrypLE (Gibco) and fixed by 4% paraformaldehyde (Sigma-Aldrich) in PBS for 30 min at room temperature. Cells were centrifuged at 500 × g for 10 minutes and resuspended in 1 ml PBS containing 2% FBS. Cells were analyzed for αMHC-GFP fluorescence using a FACS Calibur (BD-Biosciences) flow cytometer.

### Quantitative Real-time PCR

Total RNA was isolated from undifferentiated cells (day-0) or from EBs collected on various time points of differentiation protocol with the use of RNeasy mini kit (Qiagen), according to the manufacturer’s instructions. Potentially contaminating genomic DNA was digested by DNAse I (Turbo DNA-free, Ambion). The first-strand cDNA was synthesized with High Capacity cDNA Reverse Transcriptase Kit (Applied Biosystems). Real-time PCR reaction was performed using the Power SYBR® Green PCR Master Mix (Applied Biosystems) according to the manufacturer’s instructions. Primer sequences are listed in Table S3.

### Inmmunocytological staining

For ExoS_54_-Flag-TFs fusion staining, cells were fixed with 4% PFA in PBS for 15 min at room temperature. Cells were then washed 3 ×  in PBS and permeablized with 0.2% Triton X-100 in PBS. Cells were then washed 3×  in 1 × PBS with 0.05% Triton X-100 (PBST) and blocked with 1% BSA in PBST for 30 minutes. Cells were incubated with anti-Flag primary antibody for 2 hours at room temperature, then washed 3×  in PBST. Cells were then incubated with secondary antibody for 1 hour at room temperature, washed 3×  in PBST, then mounted and stained nucleus with NucBlue® Fixed Cell ReadyProbes® Reagent and examined under fluorescence microscope.

Single cardiomyocytes were isolated from embryoid body by trypLE (Gibco) and plated on gelatin-coated glass coverslips. Cells were fixed in 4% paraformaldehyde for 20 min and permeabilized with 0.1% Triton X-100 in 1 × PBS for 5–15 min at room temperature. After blocking with 10% goat serum in PBST for 1 h at room temperature, cells were stained with primary antibodies of an anti-sarcomeric α-actinin, diluted 1:100 (Sigma–Aldrich), an anti-cardiac actin, diluted 1:200 (Sigma–Aldrich) and an anti-troponin T, diluted 1:50 (Sigma–Aldrich), for 2 h at room temperature. Cells were rinsed three times with PBST and incubated for 1 h with secondary antibody (Alexa Flour 594-conjugated anti-mouse IgG, 1:200) diluted in PBST containing 10% goat serum. The slides were mounted with Vectashield containing DAPI (Vector Laboratories). Images were visualized under a Leica DMIRB inverted fluorescence microscope, captured with a DFC425 camera (Leica) and processed using the Leica Application Suite (LAS) microscope software.

### Response surface methodology

A Box-Behnken design of RSM was employed to optimize the MOI ratio of three factors (Gata4, Mef2c and Tbx5), which were investigated at 3 levels: low level (MOI = 10), high level (MOI = 50) and the center point (MOI = 30), and the experimental design used for this study was shown in Table S1. Total 15 experiments were conducted by using different MOI ratio of GMT. The corresponding responses, total fluorescence per EB (TF/EB), were calculated by Image J program. Design-Expert, version 7.0 (STAT-EASEinc, Minneapolis, USA), was used for experimental designs and statistical analysis of the experimental data. The analysis of variance (ANOVA) was used to estimate the statistical parameters.

### Contractile movement analysis

On day-12, beating clusters of cells were video recorded using a Leica DMIRB inverted microscope and Leica DFC425 camera with Micro-Manager 1.4 software at an acquisition rate of 50 frames per second (fps) for 10 seconds. After acquisition, videos were converted from TIFF stack to AVI using Image J. The AVI movies were analyzed by a cross-correlation algorithm to track the movement of pixels from frame to frame and to produce effective contractility metrics of the cardiomyocytes^63^,^64^. Isoproterenol hydrochloride (ISO), a standard stimulator of the β-adrenergic signaling cascade, and carbachol, a synthetic acetylcholine analogue acting as a cholinergic agonist, were dissolved in serum-free medium and stored according to the manufacturer’s guidelines.

### Statistical analysis

Data were analyzed by the parametric unpaired student *t* test. Values with *P* < 0.05 were considered statistically significant.

## Additional Information

**How to cite this article**: Bai, F. *et al*. Directed Differentiation of Embryonic Stem Cells into Cardiomyocytes by Bacterial Injection of Defined Transcription Factors. *Sci. Rep*. **5**, 15014; doi: 10.1038/srep15014 (2015).

## Supplementary Material

Supplementary Information

Supplementary Video S1

Supplementary Video S2

Supplementary Video S3

## Figures and Tables

**Table 1:** Strains and plasmids used in this study.

Strain and plasmid	Description	Source
*P. aeruginosa*		
PAK-J	PAK derivative with enhanced T3SS	9
ΔSTY	PAK-J deleted of *exoS*, *exoT*, *exoY*;	9
Δ8	ΔSTY deleted of *ndk*, *xcpQ*, *lasR-I*, *rhlR-I* and *popN*;	30
Δ*exsA*	PAK-J deleted of *exsA*;	11
Δ*popD*	PAK-J deleted of *popD*;	9
Plasmids		
pUCP19	*Escherichia–Pseudomonas* shuttle vector; Ap^r^	65
piExoS-Flag	pHW0224, pUCP18 containing catalytically inactive ExoS with a Flag tag; Cb^r^	66
pExoS_54_F	Promoter and N-terminal 54 aa of ExoS fused with FLAG tag in pUCP19; Cb^r^	This study
pExoS_54_F-Gata4	pExoS54F fused with *gata4* gene; Cb^r^	This study
pExoS_54_F-Mef2c	pExoS54F fused with *mef2c* gene; Cb^r^	This study
pExoS_54_F-Tbx5	pExoS54F fused with *tbx5* gene; Cb^r^	This study
