# Supplementary material for: Directed Differentiation of Embryonic Stem Cells Into Cardiomyocytes by Bacterial Injection of Defined Transcription Factors
Source: Sci Rep. 2015 Oct 9;5:15014. doi: 10.1038/srep15014 (PMC4598736; doi:10.1038/srep15014)
Supplement: Supplementary Information [file srep15014-s1.pdf]

DIRECTED DIFFERENTIATION OF EMBRYONIC STEM CELLS INTO CARDIOMYOCYTES BY BACTERIAL INJECTION OF DEFINED TRANSCRIPTION FACTORS

Fang Bai[1,2], Chae Ho Lim[3#], Jingyue Jia[1,2], Katherine Santostefano[3], Chelsey Simmons[4], Hideko Kasahara[5], Weihui Wu[1], Naohiro Terada[3], Shouguang Jin[1,2*]*

[1] State Key Laboratory of Medicinal Chemical Biology and Colleges of Pharmacy and Life Sciences, Nankai University, Tianjin, China, [2]Department of Molecular Genetics and Microbiology, [3]Department of Pathology, [4]Department of Mechanical & Aerospace Engineering, and [5]Department of Physiology and Functional Genomics, University of Florida, College of Medicine, Gainesville, Florida, United States of America

[#] Current address: Departments of Dermatology, New York University, School of Medicine, New York, New York, 10016, USA

*Corresponding authors, E-mail: terada@ufl.edu or sjin@ufl.edu

Supplementary information

I. **Supplementary Figure (Page 2-6)**

II. **Supplementary Table (Page 7-9)**

III. **Supplementary Video legends (Page 10)**

I. Supplementary Figure

Supplementary Figure 1: Elimination of residual bacteria by antibiotic treatment. mES cell line R1 was infected with Δ8 at MOI of 100 for 3 hours. Infection was terminated by washing cells with PBS and continuous growth of the mES cells on culture medium containing 20 µg/mL ciprofloxacin. After antibiotic treatment (time 0h), mES cells were washed, scraped and lysed by 0.2% Triton-X100, the lysates were used as the PCR templates to detect bacterial housekeeping gene *rpoD*. "+" indicates positive control with *P. aeruginosa* strain Δ8 lysate as the PCR template.

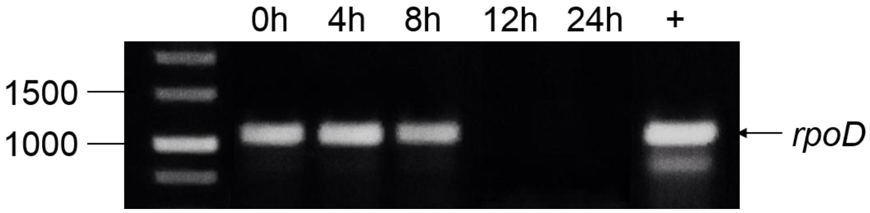

Supplementary Figure 2: Expression vector pExoS$_{54}$F. (**a**) Vector maps of the cloning plasmids used to generate the ExoS$_{54}$ fusion constructs. The ExoS$_{54}$-Flag-fusion are under the native *P. aeruginosa ExoS* gene promoter. (**b**) Sequence of the multiple cloning site located directly downstream of the ExoS$_{54}$ fragment.

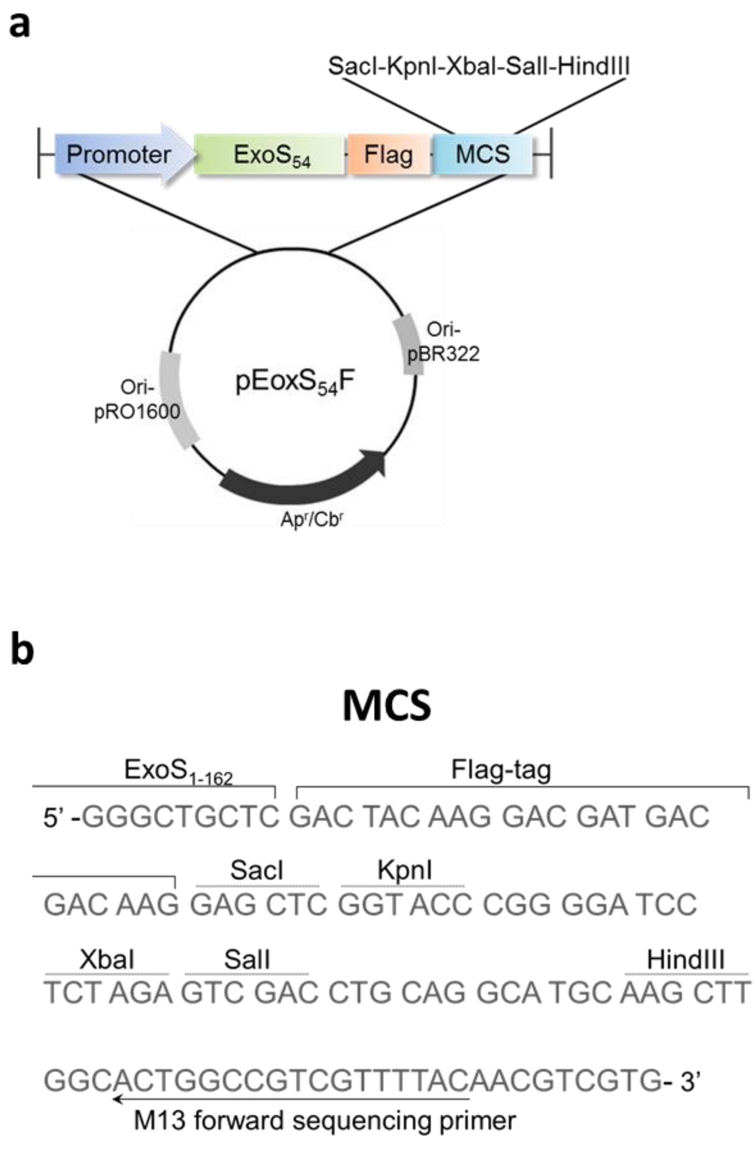

Supplementary Figure 3: Schematic representation of the standard infection procedure. *P. aeruginosa* strains were grown at 37°C in Luria broth containing carbenicillin until reaching an optical density (OD_{600}) of 0.8~1.0. Then the bacterial cells were collected, washed with PBS and diluted in cell culture medium without antibiotic. Eukaryotic cells were co-cultured with bacterial cells at various multiplicity of infection (MOI) for indicated period of time. Infection was terminated by washing cells with PBS and growing the cells on fresh cell culture medium containing 20 µg/mL ciprofloxacin.

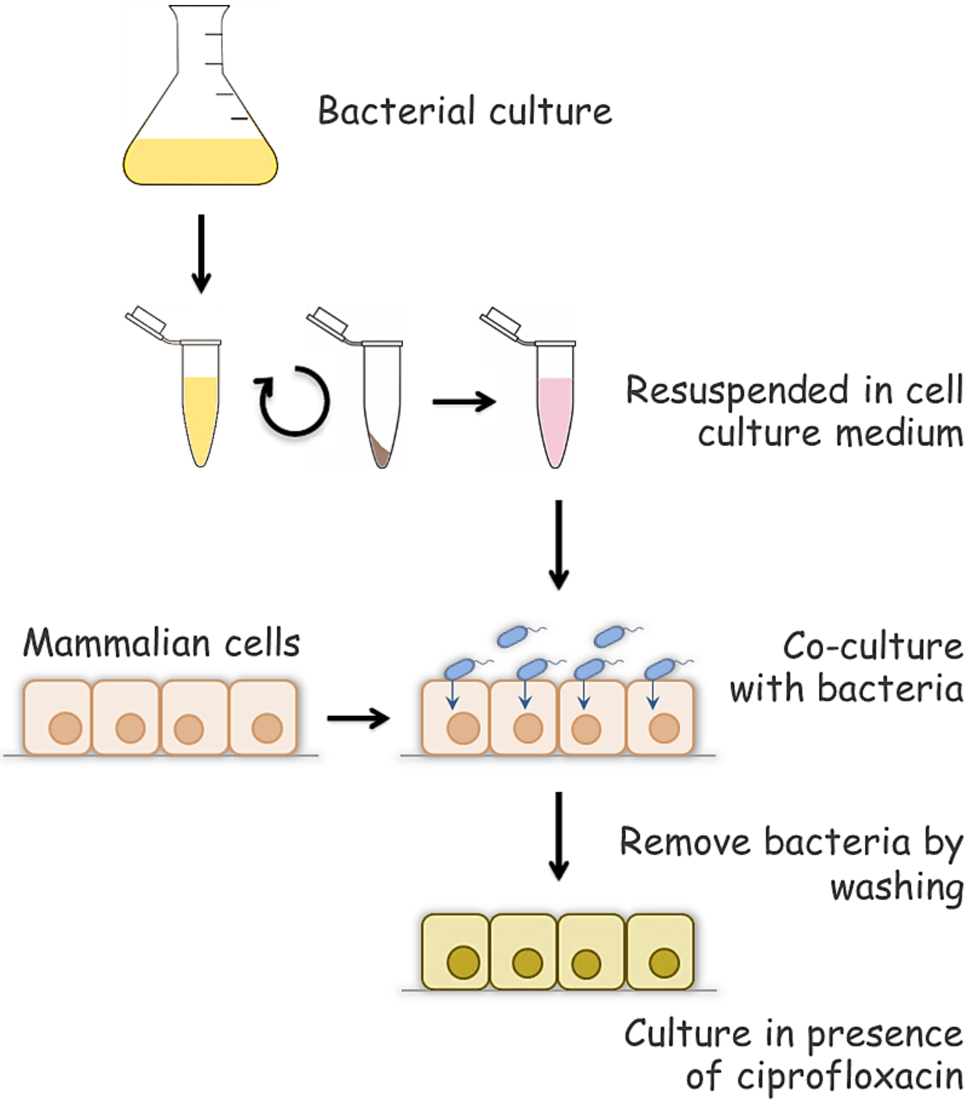

Supplementary Figure 4: (**a**) Effects of T3SS-mediated delivery of individual factors or three-factor (GMT) combination on the on GFP+ cell formation. Fluorescence intensities of EBs were measured on day-12. (**b**) Differentiating EBs were injected of the GMT (MOI of 50 per strain, 3h) once on the indicated days. Total RNAs were extracted from the EBs on day-12. Expression levels of Nkx2.5 and αMHC were measured by quantitative real-time PCR. (**c**) Images of live EBs photographed from 2 to 12 days after initiation of differentiation with/without bacterial infection (MOI 150, 3 hr).

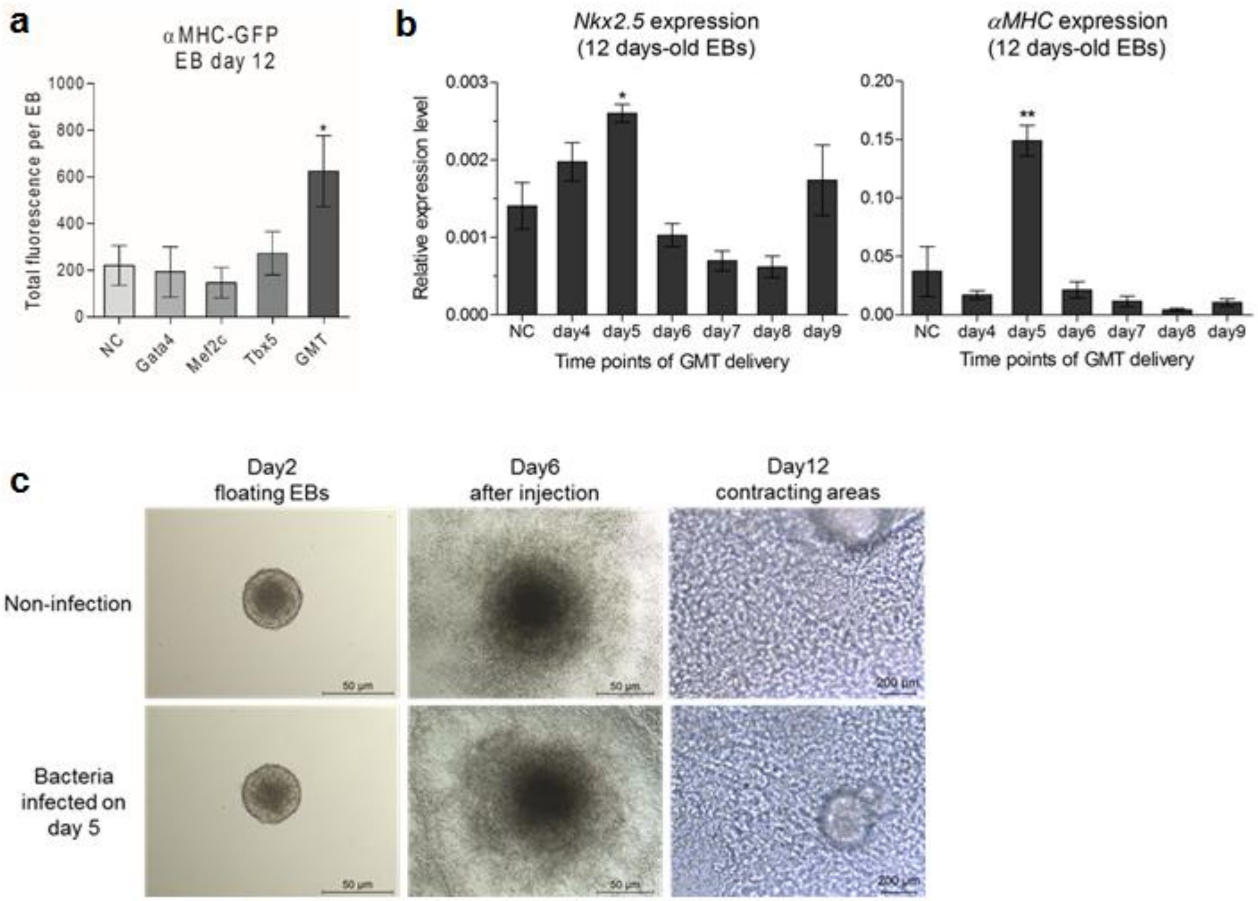

Supplementary Figure 5: Immunohistochemistry of HeLa cells following infection by Δ8/pExoS54F-Gata4, Δ8/pExoS54F-Mef2c or Δ8/pExoS54F-Tbx5 (2, 3, 4 h at MOI 50). Cells were stained with anti-Flag antibody; nuclei were stained with DAPI. Bar=50 µm.

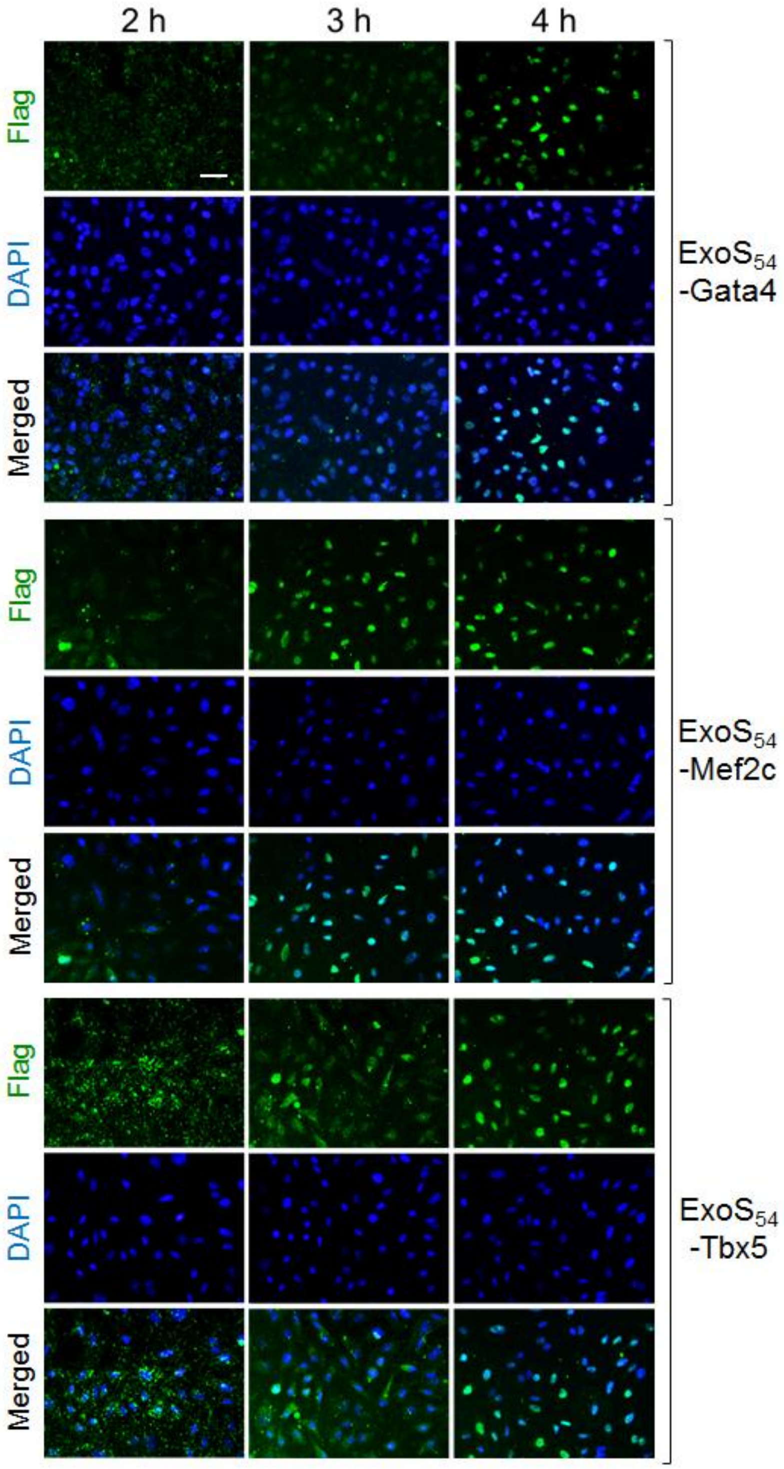

II. Supplementary Table

Supplementary Table 1

The Box-Behnken experimental design of RSM and the corresponding responses.

Run	Factors (MOI)			Response Y:TF/EB
	X_1:Gata4	X_2:Mef2c	X_3:Tbx5	
1	10	50	30	49.4
2	10	30	50	32.4
3	50	10	30	87.8
4	10	30	10	56.1
5	30	10	50	47.3
6	30	10	10	72.8
7	30	30	30	75.1
8	50	50	30	54.1
9	30	50	10	68.2
10	50	30	50	56.2
11	30	30	30	77.9
12	30	30	30	80.5
13	10	10	30	58.3
14	50	30	10	64.1
15	30	50	50	34.2

MOI: multiplicity of infection; TF/EB: total fluorescence per EB, average of n>10 EBs per condition in total.

Supplementary Table 2

ANOVA analysis of variance for response surface quadratic model in Box-Behnken experiments.

Source of variation	S.S	D.F	M.S	*F* value	*P* value	Signification
Model	3553.87	9	394.87	10.39	0.0095	*Significant
X_1-Gata4	544.50	1	544.50	14.32	0.0128	
X_2-Mef2c	454.51	1	454.51	11.95	0.0181	
X_3-Tbx5	1037.40	1	1037.40	27.28	0.0034	
X_1X_2	153.76	1	153.76	4.04	0.1005	
X_1X_3	62.41	1	62.41	1.64	0.2563	
X_2X_3	18.06	1	18.06	0.48	0.5213	
X_1^2	328.28	1	328.28	8.63	0.0323	
X_2^2	133.11	1	133.11	3.50	0.1203	
X_3^2	969.51	1	969.51	25.50	0.0039	
Residual	190.11	5	38.02			
Lack of fit	175.52	3	58.51	8.02	0.1129	Not significant
Pure error	14.59	2	7.29			
Total	3743.98	14				

S.S: sum of squares; D.F: degree of freedom; M.S: mean square.

*Statistically significant at 95% of confidence level (P value < 0.05)

Supplementary Table 3

Primers for Real Time PCR

Mouse *Gata4*	Forward	5'-TCTCACTATGGGCACAGCAG-3'
	Reverse	5'-GGGACAGCTTCAGAGCAGAC-3'
Mouse *Mef2c*	Forward	5'-ATCCCGATGCAGACGATTCAG-3'
	Reverse	5'-AACAGCACACAATCTTTGCCT-3'
Mouse *Tbx5*	Forward	5'-ACTGGCCTTAATCCCAAAACG-3'
	Reverse	5'-ACGGACCATTTGTTATCAGCAA-3'
Mouse *Brachyury*	Forward	5'-TCCCGAGACCCAGTTCATAG-3'
	Reverse	5'-TTCTTTGGCATCAAGGAAGG-3'
Mouse *dHAND*	Forward	5'-GAGAACCCCTACTTCCACGG-3'
	Reverse	5'-GACAGGGCCATACTGTAGTCG-3'
Mouse *Nkx2.5*	Forward	5'-ACATTTTACCCGGGAGCCTA-3'
	Reverse	5'-GGCTTTGTCCAGCTCCACT-3'
Mouse *α-MHC*	Forward	5'-CCAGCTAAAGGCTGAGAGGA-3'
	Reverse	5'-AGGCGTAGTCGTATGGGTTG-3'
Mouse *β-actin*	Forward	5'-TTGCTGACAGGATGCAGAAG-3'
	Reverse	5'-GTACTTGCGCTCAGGAGGAG-3'

III. Supplementary Video

Supplementary Video 1: αMHC-GFP$^+$ cell clusters in a spontaneously differentiated EB on day-12.

Supplementary Video 2: αMHC-GFP$^+$ cell clusters in a 12 days-old EB subjected to 3 rounds of GMT delivery.

Supplementary Video 3: αMHC-GFP$^+$ cell clusters in a 12 days-old EB subjected to 3 rounds of GMT delivery. Video was recorded from light field shifting to fluorescent field using a Leica DMIRB inverted microscope and Leica DFC425 camera.
